# The Evolving Role of Public Health in Medical Education

**DOI:** 10.3389/fpubh.2020.00251

**Published:** 2020-06-26

**Authors:** Ravi Rao, Melissa Hawkins, Trina Ulrich, Greta Gatlin, Guadalupe Mabry, Chaitanya Mishra

**Affiliations:** ^1^Independent Researcher, Los Angeles, CA, United States; ^2^Department of Health Studies, College of Arts and Sciences, American University, Washington, DC, United States

**Keywords:** education, public health, premedical, health equity, socio-ecological

## Abstract

Medical education in the twentieth century was largely influenced by the Flexner Report, with significant proportions of instruction dedicated to the molecular underpinnings of the pathologic pathways and minimal mention of the socio-ecological determinants of health. When examining the predominant diseases of the twenty first century landscape, widening health disparities, and significant changes in the United States healthcare system, it is imperative to view wellness and sickness in a broader public health context rather than a singular focus of the biomedical model. While undergraduate opportunities to study public health are on the rise in the United States, there is a parallel urgency for medical curricula to recognize the importance of the complex interrelated socio-ecological root causes of health, well-being, and illness. In order to reduce the risk of non-communicable diseases and increase health equity, it is necessary for medical education to integrate core public health knowledge and competencies. Contemporary health challenges require a public health approach, in addition to clinical skills, for physicians to provide equitable care. The COVID-19 pandemic further underscores the necessity to mitigate the effects of socio-ecological determinants of health. Seven key recommendations are presented from a training to practice timeline emphasizing the important linkages between medical education, socio-ecological influences on health, and public health. As the health challenges in society and communities shift, so too must training of future physicians. There is a need and an opportunity for medicine and public health to address the shared health challenges of our global society.

## Integrating Socio-Ecological Determinants of Health in Medical Education

In 1910, Abraham Flexner's report on the state of American medical education provoked a dramatic revisiting of how young physicians should be trained. The importance of science as the foundation of medical training was emphasized, while aspects of what today we call socio-ecological determinants of health were reduced. In turn, many medical schools went through significant reorganization. As with many trends in education and training, changing societal circumstances and health burdens suggest the pendulum should shift toward the other direction today with a public health perspective playing a more prominent role in medical education. Some of the intertwined factors impacting health are the widening wealth gap, social, and economic environment, the physical environment, and individual behaviors (1).

While twentieth century medical education spent significant proportions of instruction dedicated to the fundamentals of anatomy, physiology, pathology, and biochemistry, other relevant disciplines, including elements of health economics, sociology of disadvantage, community-level forces such as violence and employment opportunities, and the socio-ecological contributors across the lifespan, received minimal mention (2). Many of these factors have been the core of medical anthropology and medical sociology, but their relevance to public health is notable. When examining predominant diseases of the twenty first century landscape (e.g., cardiovascular deterioration, malignancy, chronic inflammatory conditions), the vast amount of knowledge imparted on the molecular underpinnings of the pathologic pathway greatly exceeds the training on how to view wellness and sickness in a broader public health context. “One patient at a time” may be the appropriate mindset for diagnosing some rare genetic conditions or uncommon malignancies, but becomes an inefficient and futile approach in a setting where predictable patterns of disease impact millions.

The notion of integrating socio-ecological determinants of health into medical education and healthcare systems represents a cultural shift in this country; however, the enactment of the 2010 Patient Protection and Affordable Care Act (ACA) and the establishment of the Triple Aim have recognized key public health and health equity principles. Given the impetus for evidence-based decision making in health care, public health competencies are relevant in clinical practice now more than ever.

In fact, the changing landscape and evolution of the medical field has been significantly influenced by improvements in health attributed to advancements made in the public health field. The average lifespan in the United States (US) has increased by more than 30 years in the last century, of which 25 have been attributed to public health advancements, such as reductions in vaccine-preventable illnesses, injuries and death prevented by motor-vehicle safety, and reductions in infant and maternal mortality due to advancements in family planning (3). The aging population and increasing diversity of the US population further necessitates the role of a coordinated systems approach, understanding of socio-ecological determinants of health, and cultural competence in a comprehensive medical education, all of which are cornerstones of public health.

The COVID-19 pandemic underscores the necessity for a shift in thinking about role of equity in health. In the US, the pandemic compounds existing health inequities and has illustrated the inextricable connectedness and vulnerability of society. The disproportionate impact of COVID-19 on low-income and other high-risk communities will be recognized in a variety of health outcomes long after the efforts to reduce the spread of the virus have lifted. The pandemic is a global call to action to mitigate the effects of socio-ecological determinants of health and has exposed many inadequacies in the US healthcare system, particularly community-level preventative care, for the most vulnerable populations. Although we are in the midst of this moment in history, there are already lessons learned that can be applied immediately in the healthcare setting, as well as in medical and public health curricula. Addressing socio-ecological determinants of health is a primary approach to achieving health equity (4).

While there is conceptual overlap between some of the terms used in this discussion, each refers to a different scope of knowledge and practice. Socio-ecological determinants of health refers to the entire breadth of factors that do not pertain to genetically inherited conditions; these include considerations such as poor housing, poor air/water quality, limited availability of optimal foods, and the most intractable of economic and sociological burdens that result in deprivation, and alter or increase risk to develop disease (4). Public health pertains to the health outcome and measurements thereof, and often includes interventions at community-level to reduce risk and minimize cost of caring for chronic diseases that may be ameliorated by prevention (5). Equity, defined as both a process and an outcome to reduce disparities in health (6), remains a core value in public health. Health promotion refers to both the individual-level as well as the community/national initiatives that address the socio-ecological determinants of health in order to reduce the risk of disease and injury, in order to preserve and enhance health and well-being (7). Nutrition in this context refers to the reduction of risk of diet-related illnesses.

## Activities of Future Healthcare Providers

As Westerhaus et al. (8) remarked, the current medical education system fails to “link the interplay of important biological processes with the social space their host inhabits.” Cooke et al. (9) argue further that social aspects of health, the root causes of many conditions, have been neglected in U.S. medical education for the emphasis on context-free scientific knowledge, grounded in the basic sciences. The day-to-day experience of physicians certainly incorporates scientific problem solving requiring a strong understanding of the biological sciences. Complex patients may present with atypical symptoms or consequences of polypharmacy which can only be addressed with a thorough understanding of pathophysiology and pharmacology. However, the assumption that this is the majority of the physician's workload (i.e., using cognitive efforts to apply science to a patient's chief complaint) ignores the important opportunity to improve patient's well-being and reduce risks due to socio-ecological determinants.

Future physicians may ultimately take on three roles. First, they will continue their traditional duty to treat patients at the individual level. This encompasses their medical needs as well as emotional needs for compassion and empathy. Second, as highlighted by the COVID-19 pandemic, clinicians will serve as members of a broader response to emerging threats as members of the frontline during intense short-term crises. As part of ongoing efforts to reduce the impact of chronic and communicable diseases, a public health perspective will enhance the likelihood of data sharing and other collaborative initiatives to create an organized approach to reducing their burden. Finally, healthcare providers will be increasingly be part of the digitization of healthcare information in data collection, dataset management, communication with administrative leaders, and involvement in statistical analysis. This requires quantitative rigor to examine individual health and community-level data, as well as a highly developed sense of empathy to recognize the impact of the socio-ecological determinants of health, particularly regarding inequities. Understanding statistical terminology and methodology underpins this capacity and is a core competency in public health.

## Shifting to Public Health Education

Public health as an undergraduate degree is both a relatively new concept and rapidly growing option for premedical students. The rate of obtaining a bachelor's degree in public health increased by 662% between 2003 and 2015 (10). Additionally, over the past two decades, the number of institutions offering public health degrees at the undergraduate level has increased from 45 institutions in 1992 to 176 schools that offered undergraduate public health degrees in 2012 (11). If the diseases that current (and future) physicians encounter are largely attributable to societal factors, failures of institutions to educate individuals on beneficial health practices and risk reduction approaches will illustrate that the current medical education system is limited in its ability to train physicians to handle patients with such diseases.

The landscape of premedical studies has rapidly changed since the introduction of public health as an undergraduate degree. Premedical students, in particular, may be turning to public health as an undergraduate course of study as a means to address the multitude of complex challenges facing the US healthcare system. Components of a socio-ecological medical approach, from local to global levels, and preventative care through nutrition and health promotion principles, are integral in public health undergraduate curricula and advantageous to the premedical student.

The impressive trend in the number of undergraduate students pursuing public health majors, particularly among students who are pursuing premedical studies, indicates the interest and recognition of the role of public health in clinical practice. The recent changes to the Medical College Admissions Test (MCAT) to include behavioral and social sciences also recognizes the interplay between health and society (12). Intertwined with a public health perspective is the value of nutrition education as an integral component of most health issues (13). In the US, the approximately 140 accredited medical schools require a minimum of 25 h of nutrition education; three-fourths of these medical schools fail to meet the minimal requirements (14). As we gain a greater understanding of the significance of the socio-ecological determinants of health and wellness (1), it is imperative to incorporate training in public health and nutrition into medical school requirements and curricula as well.

Alongside the recent epidemiological transition from communicable to non-communicable diseases (i.e., the plethora of chronic diseases, such as type 2 diabetes, cancer and cardiovascular disease), factors impacting health that are outside the traditional biomedical scope of physiologic diagnosis must be acknowledged in medical education. These factors, including the socio-ecological determinants of health and nutrition, continue to blur the line between public health and medicine (15). It is important to note that a public health education also involves training in community organizing, stakeholder communications, working across disciplines and with government agencies toward strategic planning and logistics and innovation, all of which are relevant to clinical practice and have been integral, most recently, in the COVID-19 response. Public health terminology also includes significant overlap with military establishments (e.g., campaigns, mobilization, surveillance, deployment), which are rooted in wartime partnership efforts. Public health also emphasizes the ability to tell compelling stories with data through effective culturally-competent health communication.

The traditional medical curricula are well-aligned with the testing that follows, but not well-aligned with the disease/illness/suffering of patients encountered in practice. Current clinical training emphasizes the scientific analysis of pathophysiology (reinforced by testing of such content). Patients are served well when in advanced stages of complex diseases, but not in the reduction of risk of such diseases or the most common chronic diseases plaguing our society.

The IOM (16) and others (17, 18) have called for medical education to integrate basic public health competencies recognizing that the traditional biomedical model fails to address factors related to the multi-factorial societal context of health and well-being. There are notable positive examples among progressive medical schools across the country and there is increased attention in medical curricula on developing skills to work with patients to reduce the risk of disease (19, 20); however, most institutions are evaluated on the basis of molecular science curricula and student performance is assessed via standardized testing on molecular science. Incorporating a public health approach to medical education has been challenging, in spite of dedicated efforts such as the identification of training providers in public health as a key measure for improving population health by the World Health Organization (21) and Centers for Disease Control and Prevention (22) and the development of the Clinical Prevention and Population Health Curriculum Framework released through the Healthy People Curriculum Task Force (23). Consistent efforts to incorporate social-ecological perspectives, nutrition education, and prevention into medical school curricula is an important next step.

## Discussion and Recommendations for Reforming Medical Education

To address the aforementioned limitations of current medical education and need for reform, seven proposals may warrant further discussion and pilot testing:

- *Premedical education*: Increase requirement of premedical students to include at least one semester of a public health course (covering disease prevention, health promotion and nutrition). In school that may not have public health department, this requirement could be fulfilled by a public health related internship or other relevant experiential learning opportunity;
- *MCAT testing*: MCAT questions should also reflect knowledge and understanding of the socio-ecological determinants of health
- *Within the first 2 years of medical school*: Include a standalone required course on public health and in nutrition within the first 2 years of medical school, including socio-ecological determinants of health frameworks and behavior change strategies. This might expand the overall class hours or be a substitute for current coursework. Concepts from this standalone course are then connected in future courses and rotations throughout medical school (immunology, surgery, obstetrics/gynecology, pediatrics, internal medicine, geriatrics, etc.). Incorporate innovative team-based and project-based learning to improve knowledge and understanding of socio-ecological determinants, disease prevention, risk reduction, and health promotion (24);
- *Post-coursework/standardized licensure testing*: Increase the number of public health and nutrition questions, including knowledge key risk factors for leading causes of death on United States Medical Licensing Examinations;
- *Research within training*: Increase or reprioritize National Institutes of Health (NIH) research funding for student and residency research programs to place greater likelihood of awards for healthy equity research and social drivers of health. Establish mechanisms supporting public health practice and academic medicine partnerships;
- *Clinical Practice*: Develop innovative payer/reimbursement mechanisms such that risk-reduction practices are financially rewarded in primary care, acknowledging the initial steps of the Patient Protection and Affordable Care Act (ACA).
- *Continuing Education*: more specialties can offer Continuing Education Units (CEUs) for completing training in public health for physicians in wide variety of practices.

Each recommendation above merits longer discussion and consideration within the fields of medicine and public health, but for purposes here, the recommendations are presented along a training-to-practice timeline. Also, this sequence focuses exclusively on physician training while numerous other disciplines are part of the provider landscape and the clinical team (e.g., nurses, physical therapist, physician associate). This proposed model is specific to the US medical education system and may follow what many countries in the world already emphasize in their medical education. In other regions of the world, physicians are often practicing community-based health given the nature of healthcare systems.

The practice of medicine centers around accurate diagnosis of illness and effective treatment of disease; public health practice complements clinical practice as it centers around preventing illness, promoting population health, and reducing inequalities in health (25). We argue that incorporating core public health competencies in medical education will make better doctors and benefit both the clinical practice and the community served. Further, as the nation faces increasingly complex interdependent health concerns and widening health inequities, such as the case with the COVID-19 pandemic, it becomes an imperative.

The need for a greater emphasis on the socio-ecological aspects of healthcare and reform in medical training is clear (25, 26). Flexner himself acknowledged that medical education must adjust in response to changing scientific, social, and economic circumstances (2). That does not, however, imply that reform is a simple endeavor (27, 28). As the needs of society shift from acute disease treatment to chronic disease prevention and management, medical training must place greater emphasis on previously underrecognized influences including socio-ecological determinants of health.

This will require changes to medical education as well as mindset shifts in the role of clinicians. However, as access to health information has increased through social media and other technological advances (i.e., telemedicine), both physicians and patients can be empowered. As such, a primary source of valid and reliable health information may be coming from periodic visits to a healthcare provider. Rather than focus exclusively on the treatment of acute illness, there is an opportunity from which patients can learn principles of public health and health promotion from their physicians. This comprehensive approach falls to the healthcare provider, regardless of specialty, which further supports that prevention, promotion, and nutrition must be incorporated in the training of physicians. Of additional importance, and lacking adequate attention, is the relevance of nutrition education surrounding the health and wellness of physicians themselves. As the burnout rate among physicians continues to rise, the need for nutrition and wellness education among physicians has also risen (29).

Medical school curricula that focus on a diagnosis and treatment perspective alone, rather than examining the complex root causes of illness, are ill-equipped to comprehensively treat patients in today's society. Medical training, on the other hand, which focuses on the interaction of biological and social relationships in determining treatment, is the future of modern medicine and training effective physicians. With the shift in the prevalence of non-communicable diseases being greater than communicable diseases, it is crucial for physicians and future physicians to recognize the influence of the socio-ecological determinants of health and to continue to intentionally blur the line between public health and medicine.

## Author Contributions

TU and MH conceived of the presented idea. GG, GM, and CM reviewed the literature and contributed to the manuscript. RR wrote sections of the manuscript. All authors discussed and contributed to manuscript revisions. All authors read and approved the submitted version.

## Conflict of Interest

The authors declare that the research was conducted in the absence of any commercial or financial relationships that could be construed as a potential conflict of interest.
